# Effect of infant stimulation on the adaptation to birth: a randomized trial*

**DOI:** 10.1590/1518-8345.2896.3176

**Published:** 2019-10-07

**Authors:** Lucy Marcela Vesga Gualdrón, María Mercedes Durán de Villalobos

**Affiliations:** 1 Universidad Nacional de Colombia, Facultad de Enfermería, Sede Bogotá D.C, CO, Colombia.

**Keywords:** Postnatal Care, Adaptation, Physical Stimulation, Health Promotion, Maternal and Child Health, Postpartum Period, Cuidado Pós-Natal, Adaptação, Estimulação Física, Promoção da Saúde, Saúde Materno-Infantil, Período Pós-Parto, Atención Postnatal, Adaptación, Estimulación Física, Promoción de la Salud, Salud Materno-Infantil, Periodo Posparto

## Abstract

**Objective:**

to measure the effect of an infant stimulation therapy (auditory, tactile, visual and vestibular) on the adaptation to postnatal life of the mother-child dyad.

**Method:**

an experimental and blind study composed of 120 dyads of first-time mothers and full-term newborns, who practiced breastfeeding. The follow-up was conducted during the first five weeks of life and the evaluation was carried at two different times.

**Results:**

the adaptive capacity was measured in two modes. *The physiological adaptive mode (activity and exercise and neonatal nutrition) and the interdependence adaptive mode (appropriate affection and proper development);* and statistically significant differences were found in favor of the experimental group. Regression models that show the collaborative relationship between mother and child, and their reciprocity in the process of adaptation were proposed.

**Conclusion:**

the early stimulation is a therapy with bidirectional effect, because it has favorable effects on the person who administers it; promotes health and prevents illness in the process of adaptation to birth; especially in contexts of vulnerability. It is recommended its teaching to mothers and its application in the home environment. This study was registered in the Australian New Zealand Clinical Trial Registry (ANZCTR) under protocol number: ACTRN12617000449336.

## Abstract (pt)

**Objetivo:**

medir o efeito de uma terapia de estimulação infantil (auditiva, tátil, visual e vestibular) na adaptação à vida pós-natal da díade mãe e filho.

**Método:**

estudo experimental, cego, que incluiu 120 díades formadas por mãe de primeiro parto e recém-nascido a termo, que praticavam amamentação materna. O acompanhamento foi realizado durante as primeiras cinco semanas de vida e a avaliação foi feita em dois momentos distintos.

**Resultados:**

a capacidade de adaptação foi medida de dois modos. *O modo adaptativo fisiológico (atividade e exercício e nutrição neonatal) e o modo adaptativo de interdependência (afeto e desenvolvimento adequados);* sendo encontradas diferenças estatisticamente significativas a favor do grupo experimental. Foram propostos modelos de regressão que mostram a relação de colaboração entre mãe e filho, e sua reciprocidade no processo de adaptação.

**Conclusão:**

a estimulação precoce é uma terapia com efeito bidirecional, pois tem efeitos favoráveis em quem a administra; promove a saúde e previne doenças no processo de adaptação ao nascimento; especialmente em contextos de vulnerabilidade. Recomenda-se seu ensino às mães e sua aplicação no ambiente doméstico. Este estudo foi registrado no *Australian New Zealand Clinical Trial Registry* (ANZCTR), sob protocolo de número: ACTRN12617000449336.

## Abstract (es)

**Objetivo:**

medir el efecto de una terapia de estimulación infantil (auditiva, táctil, visual y vestibular) en la adaptación a la vida postnatal de la diada madre e hijo.

**Método:**

estudio experimental, ciego que vinculó 120 díadas madre primeriza y neonato nacido a término que practicaban lactancia materna. El seguimiento se realizó durante las primeras cinco semanas de vida y se evaluó en dos momentos distintos.

**Resultados:**

se midió la capacidad de adaptación en dos modos. *El modo adaptativo fisiológico (actividad y ejercicio y nutrición neonatal) y el modo adaptativo interdependencia (afecto y desarrollo adecuados);* encontrando diferencias estadísticamente significativas a favor del grupo experimental. Se plantearon modelos de regresión que evidencian la relación colaborativa entre madre e hijo, y su reciprocidad en el proceso de adaptación.

**Conclusión:**

la estimulación temprana es una terapia con efecto bidireccional, pues tiene efectos favorables en quien la administra; que promueve la salud y previene la enfermedad en el proceso de adaptarse al nacimiento, especialmente en contextos de vulnerabilidad. Se recomienda su enseñanza a las madres y su aplicación en el entorno domiciliario. Este estudio se registró en *Australian New Zealand Clinical Trial Registry* (ANZCTR), con número: ACTRN12617000449336.

## Introduction

During pregnancy, the mother-child dyad begins, which is united through a symbiotic relationship in which the fetus takes from its mother what is necessary to grow and develop. After birth, the dyad changes its way of relating. This significant moment in the life of human beings is full of changes, which transform their way of interacting^(^ 1 ^)^. Mother and child face the challenge of understanding each other, and their relationship must be synchronous, since his well-being depends on her^(^ 2 ^)^. However, neither the mothers nor the newborns have the best time during the first days of postnatal life. For most people, motherhood is a wonderful experience; however, physiological, emotional and behavioral adjustments are experienced during the course of the adaptation process. The *physiological adjustments* in women show that a poor health status is associated with the mode of birth. This was the conclusion of a systematic literature review that included studies from 1996 to 2014. Sixty-two studies were selected because of their high methodological quality, which allow it to be stated that postpartum mothers have pain (headache, breast pain, backache, abdominal pain, pain after episiorrhaphy or hysterorrhaphy); fatigue and a low level of energy due to the scarce hours of rest; and they may present urinary incontinence. Thus, due to all the above factors, they have a diminished physical quality of life^(^ 3 ^)^. The newborn tests the functioning of its organs and systems, faces the challenge of feeding himself and maintaining the metabolic and hydration balance. This is a fundamental skill that will be further improved as the days go by, but if the initial difficulty is maintained, its health and growth may be adversely impacted^(^ 4 ^-^ 6 ^)^. *Emotional adjustments* in the newborn are manifested with a high level of stress^(^ 1 ^)^. A systematic review that included 15 studies of very good methodological quality evaluated the physiological and behavioral signs that newborns have in response to nociceptive stimuli associated with stress^(^ 7 ^)^. This review concluded that the responses are associated with the gestational age and are fundamentally changes in the heart rate and oxygen saturation, facial expressions, such as frowning, squeezing the eyes shut and marking the nasolabial sulcus, crying, among other signs^(^ 8 ^)^. The mother experiences a high level of anxiety, stress related to the new role, emotional sensitivity and depressive symptoms^(^ 9 ^-^ 10 ^)^. *Behavioral adjustments* are characterized by a lack of maternal skills, which increases when it is first-time mother or when the delivery is performed through a cesarean section^(^ 11 ^)^. In turn, the newborn does not know many of the environmental stimuli, so during the first days of life it has difficulty to maintain a waking state that allows it to interact with its environment, and it is not able to maintain a state of calm alertness easily, what makes the process of feeding it even more difficult^(^ 12 ^)^. The greater the difficulty of the dyad in adapting, the greater the risk of postpartum depression (PPD)^(^ 3 ^,^ 10 ^)^. PPD makes the mother-child interaction difficult and causes delays in child growth and development^(^ 3 ^,^ 10 ^-^ 11 ^,^ 13 ^)^.

Therefore, support strategies such as home visits are of great importance. These strategies are provided in different health systems with the aim of preventing habitual complications and hospital readmissions. However, developing countries such as Colombia do not provide comprehensive coverage and follow-up during this process, which is considered a highly sensitive period, and in which there is an increased risk of illness or death of the mother-child dyad^(^ 14 ^)^. These findings worry the nursing professionals, as it requires low-cost strategies that promote health and prevent diseases in this important period. Different studies show the positive effects of the early auditory, tactile, visual and vestibular stimulation therapy (ATVV), mainly in hospitalized premature newborns and their families^(^ 15 ^-^ 17 ^)^. In addition, it is presented as potentially effective when applied in the home context to benefit the process of adapting to postnatal life, since it allows the organization of the newborn behavior, which is expressed as the effective sucking ability, and that requires the hierarchical integration of social interaction systems: sensorial, autonomous, motor and behavioral^(^ 15 ^)^. It promotes growth^(^ 16 ^-^ 17 ^)^ and the emotional adjustments of the mother^(^ 17 ^)^. In this way, it is expected that the beneficial effects promote a favorable adaptation of the physiological adaptive mode (nutrition and activity and rest) and the interdependence adaptive mode (appropriate affection and proper development) of the mother-child dyads. If the newborn organizes its behavior sooner, its signals will be clearer and the relationship with its mother will be more synchronous. The effective sucking allows longer periods of neonatal sleep, contributing to the mother’s rest and the prevention of emotional problems^(^ 18 ^)^. The objective of this study was to measure the effect of the early stimulation therapy ATVV on the adaptation to postnatal life of the mother-child dyad. The adaptation was measured through the physiological adaptive (nutrition and activity and rest) and interdependence adaptive modes (appropriate affection and proper development).

## Method

**Figure 1:**
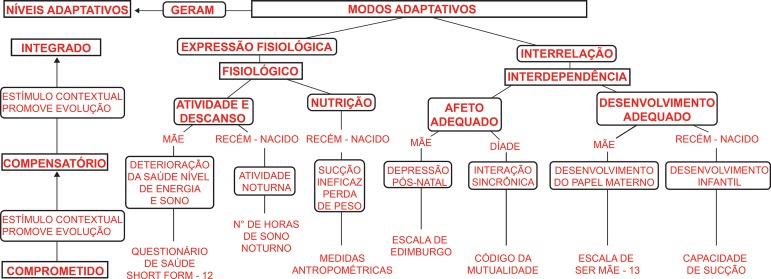
Roy’s Adaptation Model and Outcome Variables. Bogotá D.C, Colombia, 2016

Experimental study of equivalent materials determined by the mode of birth with one-to-one selection ratio, since the vaginal delivery or caesarean section can affect the outcome variables in a particular way, and repeated measurements were performed. In total, 120 dyads were approached, in the collective accommodation service of a tertiary-level hospital institution located in the city of Bogotá D.C., Colombia, from the first 24 hours after birth until the 5 weeks of postnatal life. The following criteria were considered: being first-time mothers with healthy term newborns (Apgar higher than or equal to 8 at 5 minutes) and being able to read and write. As exclusion criteria: mothers aged under 18 years, with psychological or learning disorders with previous medical diagnosis; newborns weighing less than 2,500 grams and dyads who required hospitalization during follow-up or who had stopped breastfeeding. The experimental group received the standard care of the institution and was instructed on the multisensory stimulation therapy ATVV for its home application 2 times a day, during the first two weeks. The first author trained the mothers to perform *auditory* stimulation with her own voice; *tactile* stimulation by means of sequential massage; *visual* stimulation by means of constant visual contact, and *vestibular* stimulation, cradling in their arms. This therapy follows the protocol described by American researchers in 1994, and evaluates the newborn responses of liking or disliking, which allows to adjust the intensity of the stimuli^(^ 19 ^)^. The training of the mothers followed a protocol supported by the use of audiovisual tools designed specifically for this study, which ensured the standardization of the teaching of this technique. The mothers were approached in the best possible comfort conditions and the training was offered after watching a video that described how to perform the steps of this therapy. Subsequently, the mothers were given headphones so they could perform the therapy by listening to the step-by-step instructions. The training was evaluated using a checklist. To perform the therapy at home, an audio recording was provided with the step-by-step instructions that controlled the duration and order of application of the stimuli, and ensured the reproducibility of the therapy under optimal quality conditions. The control group received the standard care of the institution and once the study follow-up was completed, this group was trained on the technique of early stimulation as a way of ethical compensation. To measure the adaptation to postnatal life, the research was guided by the Roy Adaptation Model (RAM)^(^ 20 ^)^, who states that the process can take place in Adaptive Modes. This study focused on the physiological adaptive mode (nutrition and activity and rest) and the interdependence adaptive mode (appropriate affection and proper development). *Physiological Adaptive Mode:* Through physical activity, people carry on with their daily life, relate to the environment and other beings; while rest provides moments of relaxation and repose so that the body can restore the energies. The balance between activity and sleep depends on the balance between these two processes. According to Roy’s model, situations such as pain, psychological stress and sleep disturbances affect the balance between activity and rest. Nutrition is the process by which an individual takes and assimilates the necessary food to maintain its human functioning, promote its growth and repair tissue damage or injuries. In this need, two basic processes are described, which are the digestive process and the metabolic process^(^ 20 ^)^. *Interdependence Adaptive Mode:* Appropriate affection includes the willingness and ability to give and receive love, respect, courage, education, knowledge, ability, time, loyalty. It includes the need to be nurtured in terms of care, attention, acceptance and understanding. Proper development refers to the process associated with learning and maturity. The appropriate balance between these factors influences the ability to adapt and achieve relational integrity^(^ 20 ^)^.Figure 1 shows the articulation between the concepts or outcome variables and the instruments used in the measurement in this study. All the instruments used were tested for their validity and reliability.

The mother-child dyads were observed in two moments, during the 2^nd^ and 5^th^ weeks of postnatal life. The newborns were cited 2 hours after the last intake, so that they had their sucking observed. Initially, a pediatrician performed the anthropometric measurements (weight, height and cephalic perimeter) and the mother was instructed to breastfeed her newborn. The procedure was video recorded from beginning to end. Once breastfeeding ended, a research assistant weighed the newborn again. The difference calculated in grams was attributed to the amount of milk ingested, in cubic centimeters, to determine the sucking ability.

Two specialists evaluated the video recordings to measure the effective sucking time and the number of suctions and saved them. The first minutes allowed the analysis of the Dyadic Mutuality Code. The mothers completed the questionnaires for self-reporting. The sample size was calculated by the following mathematical expression:

$$n=2{\left\{\sqrt{x_{1-\alpha }^{2}\left(k-1\right)-\left(k-2\right)+Z_{1-\beta }}\right\}}^{2}{\left(\frac{\sigma }{∆}\right)}^{2}+1$$

Where:

α: probability of committing a type I error= (0.04) β: probability of committing type II error= (0.01) σ: upper bound of the standard deviation using the values produced by the variable total score of the scale used ∆: Minimum difference between the averages of the groups to calculate the β value= (0.8σ) k: number of groups to be compared= (2). A sample composed of 60 dyads was calculated for each experimental and control group.

For the random assignment in the intervention, the authors created two blocks of 50 different random numbers between 1 and 100, using Excel’s RAND function. A block of random numbers was assigned to births by vaginal delivery and the other block to cesarean delivery births. The participants were consecutively assigned and, according to this sequence, assigned to each experimental group (EG) or control group (CG) using the corresponding table. Therefore, the same number of births by vaginal delivery and cesarean delivery births was assigned in each group. The professionals who participated as evaluators did not know the groups assignment. Mothers were informed of the beneficial effects of the early stimulation therapy on child development, but they were not informed of the effects on growth and maternal benefits.

Data analysis was carried out using the statistical package SPSS version 22. All tests were performed with a 95% confidence interval and two main stages were followed: one to estimate the differences between the experimental and control groups using the Mann-Whitney U test; and the other one to estimate the differences between each group at the two assessment moments using the Wilcoxon test, considering a non-normal distribution of the data. Regression models were proposed^(^ 21 ^)^. Additionally, G*Power 3.1.9.2 free software was used to calculate the effect size (ES), estimated by the Cohen’s D values using the differences between means. The research was approved by the ethics committee of the nursing faculty of the National University of Colombia and by the research ethics committee of the health institution where the study was conducted.

## Results

**Figure 2:**
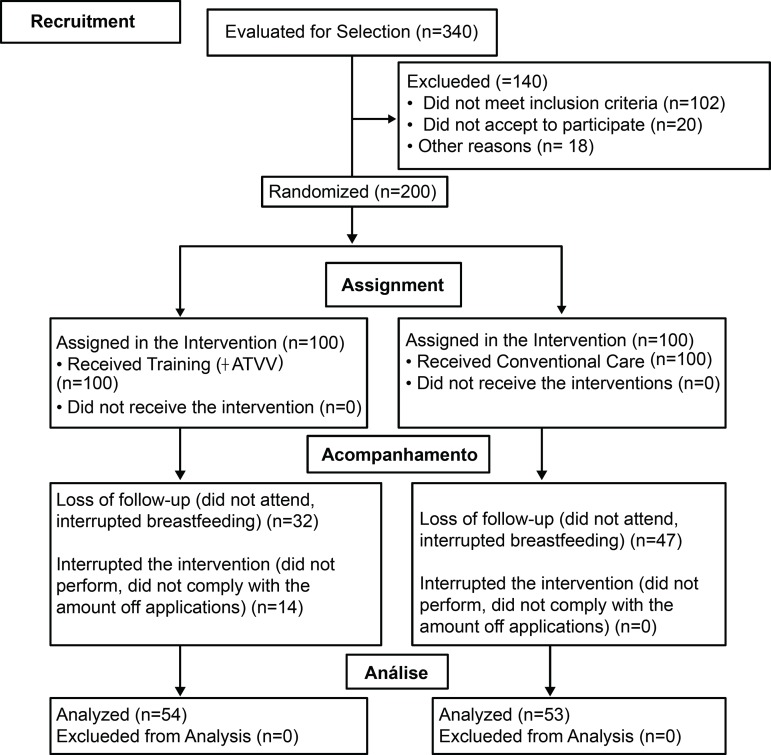
Flow chart of participants in accordance with the statement of the Consolidated Standards of Reporting Trials (CONSORT*). Bogotá D.C, Colombia, 2016 *CONSORT: Consolidated Standards of Reporting Trials; †ATVV: Auditory, Tactile, Visual and Vestibular Stimulation

The fieldwork was carried out from July 19 to November 27, 2016, at a Hospital in the city of Bogotá, D.C., Colombia. Taking into account the loss of participants, a larger number of participants was approached. However, despite the efforts undertaken, it was not possible to count on the estimated sample size at the end of the follow-up. Figure 2 shows the flow chart.

The greatest damages were observed in those mothers who underwent cesarean section, since mobility restriction in the days after delivery was a recurrent cause of their nonattendance at the evaluation meetings. Table 1 shows the description of the sociodemographic characteristics of participants and reveals that they are mothers with a low level of education, employed in low-skilled jobs, with a family income of around 320 dollars per month, that is, equivalent to a little more than one local minimum wage. The average length of labor was 8 hours.

**Table 1:** Socioeconomic characteristics of mothers. Bogotá D.C., Colombia, 2016

Control Group (n=53)			Experimental Group (n=54)		T^†^ (<0.05)
Variables	Mean	SD*	Mean	SD*	
Age	25.2	6.2	24.3	4.2	(0.9)
Monthly income	1062115,4 COP^‡^	792566,8 COP^‡^	1055090,9 COP^‡^	683874,8 COP^‡^	(0.4)
Years of study	12.9	2.5	13.3	2.1	(0.3)
Length of Labor in Hours	7.5	4.9	8.1	5.5	(0.6)

* SD = Standard Deviation;

† T = Mann-Whitney U test;

‡ COP = Colombian Peso

The newborns exhibited a similar distribution, according to sex and APGAR scores. Other variables describing the physiological maturation of the newborns are shown in Table 2, which reveals in detail that the anthropometric measures and the gestational age determined by the pediatrician using the Ballard test at birth are similar between the groups.

**Table 2:** Characteristics of newborns at birth. Bogotá D.C, Colombia, 2016

Control Group n=53					Experimental Group n=54				T^§^ <0.05
Variables	Min*	Máx^†^	Mean	SD^‡^	Min*	Máx^†^	Mean	SD^‡^	
Weight	2560	4100	3152.7	361.4	2550	4080	3077.3	362.9	(0.3)
Height	47	55	50.7	1.8	47	53	50.3	1.4	(0.2)
Head Circumference	31	36	33.9	1.2	31.5	36	33.7	1.1	(0.4)
Gestational age	37	40	39.0	0.9	37	40	38.9	1.0	(0.9)
Apgar at minute	7	8			6	9			(0.09)
Apgar at 5 minutes	8	9			8	9			(0.09)

* Min = Minimum;

† Max = Maximum;

‡ SD = Standard Deviation;

§ T = Mann-Whitney U test

The analyses show that the groups are homogeneous between themselves, an indispensable feature to evaluate the effect of the early stimulation therapy. Nonparametric tests were performed to evaluate the differences between the experimental and control groups at the two assessment moments. *In the physiological adaptive mode: Activity and rest*, the number of hours of night sleep of the mother and newborn was measured and no statistically significant difference was found by the Mann-Whitney U test (*p:* 0.268) at the first measurement point. In contrast, statistically significant differences were found at the second measurement point (*p:* 0.034) CG (Mean: 7.82 hours, SD: 1.152) vs EG (Mean: 8.33 hours, SD: 1.194) (Cohen’s D: 0.908), with a large effect size^(^ 22 ^)^. Similar results were found in the second measurement of the perception of the mother’s general health, measured using the SF-12 scale (*p:* 0.001) CG (Mean: 79.91 SD: 15.55) vs EG (Mean: 89.44, SD: 12.689) (Cohen’s D: 0.736), with a large effect size^(^ 22 ^)^.

*In the physiological adaptive mode: nutrition*, the results show a favorable effect of the therapy on neonatal growth and sucking ability. With regard to weight, height and growth in head circumference in newborns, the Mann-Whitney U test showed statistically significant differences. Weight gain at the first measurement point (*p:* 0.009) CG (Mean: 4.34 grams, SD: 16.95) vs EG (Mean: 12.69 grams, SD: 13.41) (Cohen’s D: 0.54) showed a large effect size^(^ 22 ^)^. In the second measurement (*p:* 0.000) GC (Mean: 29.10 grams, SD: 8.01) vs GE (Mean: 33.29 grams, SD: 7.81) (Cohen’s D: 0.53), it was observed a large effect size^(^ 22 ^)^. Height and head circumference showed differences in the second measurement. Height measurement (*p:* 0.025) CG (Mean: 0.084 cm, SD: 0.05) vs EG (Mean: 0.109 cm, SD: 0.05) (Cohen’s D: 0.4) showed a small effect size^(^ 22 ^)^. Head circumference measurement (*p:* 0.041) CG (Mean: 0.0865 cm, SD: 0.029) vs EG (Mean: 0.1007 cm, SD: 0.31) (Cohen’s D: 0.78) showed a large effect size^(^ 22 ^)^.

*In the interdependence adaptive mode: Appropriate Affection and Proper Development*, the statistical tests show that the ATVV stimulation prevents PPD, measured by the Edinburgh Scale, Mann-Whitney U test (*p:* 0.000) CG (Mean: 6.91, SD: 4.11) vs EG (Mean: 4.31, SD: 2.83) (Cohen’s D: 0.632), with a medium effect size^(^ 22 ^)^. Its also improves the synchronism in the mother-child interaction and decreases the risk of stress related to maternal role, measured by the BaM-13 scale, Mann-Whitney U test (*p:* 0.001) CG (Mean: 9.6, SD: 5.29) vs EG (Mean: 6.19, SD: 4.12) (Cohen’s D: 0.662), with a medium effect size^(^ 22 ^)^. These results were amply described in other research articles.

The variables were submitted to a logistic regression analysis, including the different variables considered as important in the literature, which were accessed in the statistical program by pressing the enter key. These include: mode of birth, monthly income, level of education, sex, weight and gestational age of the newborn, complications during pregnancy, physical pain in the postpartum period, perception of the mother’s health, her physical function and performance of her role, emotional state of the mother, amount of night sleep of the mother and child, synchronism in their interrelation, age and feeding of the newborn. Several models were analyzed including the following aspects: 1) the correct classification percentage of the model, 2) the Chi square test, 3) the decrease in the deviation (-2 times the Likelihood Logarithm). Finally, a regression model to explain the risk of stress related to maternal role measured in the second week of postnatal life was proposed with the following properties: sensitivity of 69.8%, specificity of 92.2%; total adjustment percentage of 83.2%; Chi square: 45.822 (*p:* 0.000), which ensures that independent variables can explain the response variable; -2 times the Likelihood Logarithm: 98.363. These characteristics explain 47.1% (0.471: Nagelkerke’s R squared) of variance of the risk of maternal stress. These data are shown in Table 3.

**Table 3:** Logistic regression model: stress related to maternal role in the 2nd week. Bogotá D.C, Colombia, 2016

Variables in the equation						
1^st^ Stage	B	Standard error	Wald	df	Sig.	Exp(B)
Age of the mother	-0.116	0.055	4.505	1	(0.034)	0.890
Type of feeding of the RN*	1.519	0.681	4.970	1	(0.026)	4.566
PPD Scale (EPDS)^†^	0.442	0.097	20.819	1	(0.000)	1.556
Constant	-0.754	1.299	0.337	1	(0.562)	0.471

* Type of feeding of the NB = (0: Breastfeeding), (1: Mixed Feeding);

† PPD Scale (EPDS) =Edinburgh Scale

These characteristics allow us to select the previous regression model and qualify it as a good model to explain the risk of stress related to maternal role using 3 independent variables. It is concluded that, the older the mother, the lower the risk of stress related to maternal role (OR: 0.890, *p:* 0.034). In addition, the use of a nursing bottle to feed the newborn increases the risk (OR: 4.566, *p:* 0.026), and high scores on the scale of postnatal depression increases the risk of stress related to maternal role (OR: 1.556, *p:* 0.000). Another remarkable finding is that belonging to the experimental or control group has no influence on the response variable. This result is consistent with the tests to estimate the differences between groups, used for measurements during the first 2 weeks of postnatal life.

Based on Roy’s adaptation model, it is confirmed that the mother-child dyad is an *Integrated System*, which cannot be interpreted in isolation, since the conditions that affect one also affect the other, so its adaptation must be understood jointly.

## Discussion

Despite the efforts of the researchers to carry out the follow-up of the mother-child dyads, there was a large loss of participants during this study. Living conditions that are typical in a country in conflict, such as internal displacement and insecurity, which makes it difficult to use other forms of follow-up, such as home follow-up. It is possible that these findings are due to the pain and mobility limitations of mothers in using public transport during this short period, as well as the cultural practice of moving to the maternal home for quarantine care, even when it is necessary to move to another city. In view of the results in the Physiological Adaptive Mode, this study supports the results previously found by authors from Korea^(^ 17 ^)^ and Colombia^(^ 23 ^)^ on the positive effect of the stimulation therapy on growth; as well as the favorable effect on the sucking ability mentioned in previous studies conducted in the United States^(^ 16 ^,^ 24 ^-^ 25 ^)^. Regarding the findings of this study on activity and exercise, no other authors who have applied this therapy before in the infantile and maternal sleep and in the general health of the woman in the puerperium were found. The results of the effect of this therapy in the Interdependence Adaptive Mode coincide with those found in studies conducted with mothers of extremely premature newborns, comparing with the results of PPD^(^ 15 ^)^. No study measuring the effect of ATVV therapy on stress related to maternal role was found.

On the regression model, it is important to analyze the influence of the type of feeding, since the sucking ability is the expression of the organization of newborn behavior. The establishment of breastfeeding depends on the vigorous and continuous sucking of the newborn from its birth, which can positively or negatively affect the production of breast milk^(^ 26 ^-^ 27 ^)^, and indirectly affect the amount of milk that the newborn can to suck. When this process does not take place in a harmonious way, mothers make use of bottle feeding and breast milk substitutes^(^ 28 ^)^. Therefore, the evidence shows that there is a negative relationship between bottle-feeding and the sucking ability of the newborn and the establishment of breastfeeding, which delays the organization of behavior. In this way, having a slower organization of behavior leads to a greater maternal emotional imbalance during the first days of life. These findings are consistent with those of previous studies that point out the relationship between breastfeeding difficulties and an increased risk of PPD^(^ 28 ^)^, but the evidence in this regard is still inconclusive.

This study recognizes among its limitations the lack of budget and the large loss of participants during follow-up, which caused the extension of the data collection period to attenuate it. Additionally, no strategies were available to control the application of home therapy doses. Other studies have used tools such as diaries or written records of participants, and no data on the instrumentation used during vaginal deliveries were obtained, which may have some influence on aspects such as the general health of mothers in the postpartum period.

## Conclusions

The multimodal stimulation therapy ATVV applied by mothers to their healthy newborns, at home, generates important benefits especially in contexts of vulnerability and resource shortages. The results show a positive effect on the sooner neurobehavioral organization, demonstrated in the sucking ability of the newborn, as well as in the favorable evolution of anthropometric measures (weight, height and head circumference). These findings are relevant to the professional nursing practice, since its effects allow the application of a strategy with low cost and with no known risks for a wide range nutritional risk conditions for the newborn. Another important aspect is the emotional adjustment of the mother, since the therapy is shown to be an effective tool in the prevention of emotional damages such as the stress related to maternal role and PPD. The latter is considered a major problem for the women’s health worldwide. Therefore, this intervention should be continued and promoted in order to facilitate the adaptation of the mother-child dyad to postnatal life.

The application of early stimulation therapies, such as ATVV therapy in teaching for healthy children, which should be provided by nurses to the mothers, allows the practice of evidence-based nursing and enriches the tools for health promotion and disease prevention.
